# Emotional Coping Differences Among Breast Cancer Patients From an Online Support Group: A Cross-Sectional Study

**DOI:** 10.2196/jmir.2831

**Published:** 2014-02-05

**Authors:** Anika Batenburg, Enny Das

**Affiliations:** ^1^VU University AmsterdamDepartment of Communication ScienceAmsterdamNetherlands; ^2^Radboud University NijmegenCentre for Language StudiesNijmegenNetherlands

**Keywords:** Internet, support groups, self-help groups, social support, online systems, emotions, coping style

## Abstract

**Background:**

Due to mixed findings in research on the effect of online peer-to-peer support on psychological well-being, there is a need for studies explaining why and when online support communities are beneficial for cancer patients.

**Objective:**

Previous studies have typically not taken into account individual coping differences, despite the fact that patients have different strategies to cope with cancer-related emotions. In the current study, it was predicted that the effects of online support group participation would partly depend on patients’ ability to cope with thoughts and emotions regarding the illness.

**Methods:**

For this study, 184 Dutch breast cancer patients filled out a questionnaire assessing activity within a peer-led online support community, coping with emotions and thoughts regarding the illness (cognitive avoidance, emotional processing, and expression) and psychological well-being (depression, breast cancer-related concerns, and emotional well-being). Of these, 163 patients were visiting an online peer-led support community.

**Results:**

Results showed interactions of the intensity of support group participation and coping style on psychological well-being. Specifically, we found an interaction of online activity and emotional expression on depression (beta=–.17, *P*=.030), a marginally significant interaction of online activity and emotional expression on emotional well-being (beta=.14, *P*=.089), and an interaction of online activity and cognitive avoidance on breast cancer–related concerns (beta=.15, *P*=.027). For patients who actively dealt with their emotions and thoughts, active online support group participation was positively related to psychological well-being. For patients high on avoidance of illness-related thoughts or low on emotional expression, active participation was negatively related to measures of well-being.

**Conclusions:**

The current study revealed the role of individual differences in coping in online support group participation. Results suggest that breast cancer patients’ ability to cope with emotions and thoughts regarding the illness influence the relationship between online support group participation and psychological well-being.

## Introduction

As in other countries, the number of newly diagnosed breast cancer patients in the Netherlands has increased in recent years (up 18% between 2000 and 2010; 13,357 new patients in 2010) and is expected to rise because of the extended life span of the population, the early detection of breast cancer, and the increasing effectiveness of treatment [1]. A greater number of patients are turning to the Internet for support and for information about health-related issues [2,3]. Research has shown that breast cancer patients are among the most active seekers of online social support compared to other patient groups [4]. Several studies have set out to reveal the effects of online peer support on psychological well-being, showing mixed findings [5-7]. For example, some studies revealed positive outcomes, such as decreased depression, reactions to pain, cancer-related trauma, and distress [8-10]. Other studies found no increase in well-being [11,12] or reported negative associations or effects [13,14].

These mixed findings may stem from the fact that most outcome studies assess online support interventions set up by professionals. These interventions often include other therapeutic elements besides peer support, which makes it hard to disentangle the effects of solely the online peer support [6]. Studies testing the effects of peer-led online support communities are underrepresented in the literature, even though these communities are common and easily accessible online. One reason might be that testing the effects of participating in peer-led communities is complicated due to its uncontrolled setting. Therefore, studies examining these peer-led groups are largely qualitative or descriptive in nature and suggest the presence of empowering processes, such as emotional and informational support, emotional expression, advice, recognition, understanding, and insight [15-19]. However, follow-up research is needed to assess the relationship between these therapeutic processes and health outcomes. Therefore, the first goal of our research was to provide a quantitative test of the relationship between participation and well-being in peer-led support groups.

Another reason for mixed findings in outcome studies is the individual differences between patients. Although descriptive studies suggest therapeutic processes at work within online support communities, these processes may not apply equally to all patients. Some patients only read stories from others, while others share experiences, thoughts, emotions, ask questions, or support others [20]. Although patients have the opportunity to disclose their feelings and read about the experiences and emotions of others online, not every patient approaches emotions equally. Previous descriptive studies show a large variety in content of online messages, from emotional experiences to illness to unrelated chitchat [21,22]. Furthermore, studies using a word counting program have shown that there are variations in individual writing styles and that these variations are related to health outcomes. For example, greater expression of negative emotions, cognitive processing, and lower expression of health-related concerns was positively associated with quality of life variables [11], and words suggestive of learning or understanding improved emotional well-being, functional well-being, health self-efficacy, and reduced negative mood [23]. Thus far, individual differences in how patients cope with thoughts and emotions have not been connected to potential effects of support group participation. A second goal of the present study therefore was to examine the relationship between peer-led support group participation, individual differences in coping behavior, and measures of well-being.

The intensity of support group use and how patients disclose thoughts and emotions depend to a large extent on individual coping styles. From offline studies, we know that some breast cancer patients approach the illness actively by processing and expressing their emotions [24], while others try to avoid all thoughts and emotions related to the illness [25]. The tendency to approach thoughts and emotions can be categorized on three levels of emotional engagement: (1) cognitive avoidance: the patient tries to avoid all thoughts and emotions related to the illness [26], (2) emotional processing: the attempt to acknowledge and understand feelings, but not necessarily the expression of emotions [24,27], and (3) emotional expression: individuals not only acknowledge their emotions but also allow themselves to express them [24]. Several studies have shown that the use of a cognitive avoidant coping style is negatively related to adjustment to cancer [28], health status [29], and is positively related to higher distress levels [25,30]. In contrast, actively dealing with thoughts and emotions regarding breast cancer showed a positive relationship with psychological and physical well-being, such as decreased depressive symptoms, distress, increased vigor, improved perceived health status, and fewer medical appointments [24,31-33]. Therefore, consistent with the coping literature, we expect that patients who score relatively high on avoidance coping will report a lower sense of well-being than patients who show less avoidant behavior. In contrast, patients who actively approach their emotions are expected to report a better sense of well-being than patients who approach emotions less actively.

Although one would expect that patients participating in an online support community are willing to confront illness-related information and therefore cope with their emotions and thoughts quite actively, a certain degree of online avoidance, processing, and expression might influence patients’ well-being. Based on previous findings, it seems plausible to assume that active online support group participation may positively affect well-being in particular for patients who score low on cognitive avoidance and high on emotional processing and expression coping, because these patients are likely to benefit most from interactions with peers. Therefore, we suggest that coping styles are not only directly related to psychological well-being, but they also moderate the relationship between online support group participation and psychological well-being.

Due to the lack of studies on peer-led online support groups, we conducted a study on Dutch online communities set up by patients and former patients. Because previous mixed findings in research might partly have been caused by individual differences, this is the first attempt to include patients’ coping style regarding illness-related thoughts and emotions. Although psychological research has extensively shown the relationship between coping styles and health outcomes [25,27-33], to our knowledge no study has assessed this relationship in the context of online peer support. We conducted our online study among Dutch breast cancer patients to measure the intensity of their online support group participation, coping style, cognitive avoidance, emotional processing, and emotional expression, as well as three measures of well-being generally associated with breast cancer diagnosis: emotional well-being, depression, and breast cancer-related concerns [34-36]. We also included additional factors in our analyses often associated with the psychological well-being of breast cancer patients, such as social support from family and friends [37,38], illness stage, and professional psychological help received. We hypothesized that active online support group participation would be positively related to emotional well-being and negatively related to depression and breast cancer-related concerns in particular for patients who scored low on cognitive avoidance and high on emotional processing and expression.

## Methods

### Participants and Procedure

We used Google to identify all online support communities for breast cancer patients in the Netherlands. Criteria for inclusion were that (1) the website was in the Dutch language, (2) the support group (sometimes part of a more extensive website) was designed as a peer-led message board available 24/7, and (3) the discussion board was still active (the last month’s new messages had been posted). With approval of the website owners, a request to participate in an online survey about breast cancer patients’ Internet use was posted on seven support websites (June 2011). This survey was part of a more extensive research project on online peer support among Dutch breast cancer patients. The research was carried out in accordance with the American Psychological Association’s ethics guidelines [39] and complies with European Union legislation [40] and Dutch legislation [41] on data protection.

The introduction page (ie, the first page of the survey) included the length and purpose of the survey, ensured anonymity, and contact information of the investigator (in case participants had any questions). The first page of the survey was viewed 311 times, and 184 Dutch breast cancer patients filled out the questionnaire (182 females and 2 males). Response rates are unknown because we had no access to page views of the participating websites. The online survey tool tracked IP (Internet protocol) addresses to prevent users from re-taking the survey. Responses to questions were obligatory, but participants were provided with an “I don’t know” or “not applicable” option.

Since males were underrepresented, we decided to exclude them from data analysis. Another 7 participants were excluded, due to extreme responses on one of the dependent variables (SD>3). Therefore, 175 participants were included in data analysis. Table 1 shows the demographics and patient characteristics of the study sample. Table 2 shows the average use of the peer support message board.

**Table 1:** Demographics and health characteristics (N=175).

Characteristics			n	%
**Age**				
	Mean (SD)		48.09 (9.04)	
	Minimum		23	
	Maximum		71	
**Education** ^a^				
	Elementary school		5	2.9
	**Secondary education**			
		Low	29	16.6
		Middle	15	8.6
		High	2	1.1
	**Tertiary education**			
		Low^b^	10	5.7
		Middle	47	26.9
		High	55	31.4
		Scientific degree	12	6.9
**Working status**				
	Not working		90	51.4
	Working		85	48.6
**Illness stage**				
	No cancer cells at the moment		27	15.4
	Stage I (tumor smaller than 2 cm, no metastases to the lymph nodes)		41	23.4
	Stage II (metastases to the lymph nodes in the armpit, or a tumor larger than 2 cm with no metastases)		42	24.0
	Stage III (metastases to multiple lymph nodes or other lymph nodes)		25	14.3
	Stage IV (metastases to other body parts)		16	9.1
	Unknown		24	13.7
**Psychological help during period of illness**				
	Yes		69	39.4
	No		106	60.6

^a^Levels within the Dutch education system: education is divided over three schools for different age groups, which are divided in streams for different educational levels.

^b^Dutch educational structure ^“^LBO/LTS” existed until 1992.

**Table 2:** Use of the online support community (N=175).

Frequency of use		n	%
Not visiting an online BC support message board		12	6.9
**Frequency of visits (n=163)**			
	<1 per month	19	11.7
	Approximately once a month	12	7.4
	Multiple times per month	12	7.4
	Approximately once a week	17	10.4
	Multiple times per week	22	13.5
	Approximately once a day	44	27.0
	Multiple times per day	37	22.7
**Frequency of posts the last 4 weeks (n=163)**			
	None	61	37.4
	≤1 per week	39	23.9
	Multiple posts per week, but not every day	45	27.6
	Every day one post or more	18	11.0
**Forum contribution (n=163)**			
	I only read posts from others	36	22.1
	I reacted on post(s) of someone else	28	17.1
	I started a new topic or asked a question	18	11.0
	I both started a new topic or asked a question AND I reacted on post(s) of another	81	49.7
**Average length of visits (n=163)**			
	<10 minutes	61	37.4
	10-30 minutes	71	43.6
	30 minutes to 1 hour	22	13.5
	>1 hour	9	5.5

### Measurements

#### Online Support Group Participation

The intensity of online support group participation was assessed by four different questions regarding frequency of visits, average length of visits, contribution, and frequency of posts in the last 4 weeks (cf [42]). Frequency of visits was assessed on a 7-point scale; the other items were assessed on a 4-point scale (Table 2). To merge these different scales into one index, all items were transformed into Z scores (Cronbach alpha=.77).

#### Emotional Coping

The Dutch mini-MAC [24] was used to assess *cognitive avoidance* (4 items, eg, “I try not to think about my illness”). Participants rated on a 4-point scale if the statements applied to them. Ratings were summed and averaged across items. Higher scores indicated that the coping style of cognitive avoidance applied to them. The scale was internally consistent (Cronbach alpha=.82).

The Emotional Approach Coping scale [22] was used to measure *emotional processing* and *emotional expression*. Four items measured emotional expression (eg, “I take the time to express my emotions”; Cronbach alpha=.86). Another four items measured emotional processing (eg, “I realize that my feelings are justified and important”; Cronbach alpha=.69). Participants rated on a 4-point scale if the statements applied to them. Ratings were summed and averaged across items. The variables indicating the intensity of support group participation and coping styles were unrelated.

### Psychological Well-Being

Three different scales measured psychological well-being. First we measured depression using the Center for Epidemiological Studies Depression Scale (CES-D10) [43]. The scale consisted of 10 items (eg, “I felt that everything I did took me quite a lot of effort”). Participants rated on a 4-point scale if the statements applied to them the last week, from “less than one day” to “5 to 7 days”. Ratings were summed and averaged across items. Higher scores indicated more depression-related thoughts. The scale was internally consistent: Cronbach alpha=.72. Additionally, we measured breast cancer-related concerns [44] with 28 items (eg, “Are you concerned that your friends will avoid you?”; Cronbach alpha=.89). Participants answered these questions on a 5-point scale, ranging from “Not at all” to “Totally”. Higher scores indicated more concerns regarding the illness. Emotional well-being was measured according to 6 items from the Functional Assessment of Chronic Illness Therapy questionnaire (FACIT-B) [45] (Cronbach alpha=.81). An item example is “I feel sad”. Respondents rated on a 5-point scale if the statements applied to them, ranging from “Not at all” to “Totally”. Higher scores indicated a better sense of emotional well-being.

### Control Variables

Last, we measured participants’ age, education level, current working status (ie, if they were currently working), illness stage (the standard four phases in breast cancer [46]), psychological help (ie, if they received psychological help from a professional), and offline social support (based on the six “Social well-being” items from the FACIT-B [45]). Items referring to support from friends were adjusted into items that clearly referred to their offline friends, not online peers. Respondents rated on a 5-point scale if the statements applied to them, ranging from “Not at all” to “Totally” (Cronbach alpha=.94).

## Results

### Correlations

Tables 3 and 4 show the correlations between all variables. Cognitive avoidance coping was associated with all three psychological well-being variables: positively with depression and breast cancer–related concerns and negatively with emotional well-being. Emotional processing was not related to psychological well-being. Emotional expression was negatively related to depression. No direct associations between support group participation and one of the psychological well-being variables were found.

We tested the hypothesized relationship between online support group participation, coping styles, and psychological well-being with regression analyses. All independent variables were standardized into Z scores to meet the requirements to perform regression analyses and to compute interaction terms. In addition, every model included the covariates that were significantly correlated to the dependent variable (ie, depression, emotional well-being, breast cancer-related concerns). We examined interactions for participants with relative low scores (1 SD below the mean of the standardized score) and for participants with relative high scores (1 SD above the mean of the standardized score) on the continuous indices measuring intensity of online participation and coping styles (see [47] for this regression analysis).

**Table 3:** Means, standard deviations, and intercorrelations of independent variables, illness stage, age, and dependent variables.

Variables		n	M	SD	1	2	3	4	5	6
1	Support group participation^a^	163	–0.01	0.78	–					
2	Cognitive avoidance	175	1.95	0.60	.03	–				
3	Emotional processing	174	2.86	0.50	.01	–.10	–			
4	Emotional expression	174	2.79	0.54	.07	–.16^d^	.57^c^	–		
5	Illness stage	151	2.75	1.23	.23^c^	.06	–.08	–.09	–	
6	Age	175	48.09	9.04	–.24^c^	–.02	–.03	.02	–.01	–
7	Education	175	6.40	1.93	.01	–.13	.09	.03	.11	–.26^c^
8	Working status^b^	175	0.49	0.81	–0.12	.01	–.08	–.14	–.13	–.20^c^
9	Offline social support	175	3.78	0.60	.03	–.18	–.02	.18^d^	–.03	–.05
10	Psychological help^b^	175	0.39	0.49	–.06	.11	.15	–.04	.00	–.22^c^
11	Depression	175	1.83	0.45	.01	.36^c^	.05	–.15^d^	.04	–.06
12	Breast cancer-related concerns	175	2.64	0.55	.08	.35^c^	.03	–.02	.07	–.18^d^
13	Emotional well-being	175	3.53	0.77	–.02	–.47^c^	–.06	.06	–.20^d^	.04

^a^Standardized into Z scores.

^b^Coded 0=no, 1=yes.

^c^Correlations significant at the .01 level.

^d^Correlations significant at the .05 level.

**Table 4:** Means, standard deviations, and intercorrelations of education level, working status, psychological, and offline support, and dependent variables.

Variables		n	M	SD	7	8	9	10	11	12
1	Support group participation^a^	163	–0.01	0.78						
2	Cognitive avoidance	175	1.95	0.60						
3	Emotional processing	174	2.86	0.50						
4	Emotional expression	174	2.79	0.54						
5	Illness stage	151	2.75	1.23						
6	Age	175	48.09	9.04						
7	Education	175	6.40	1.93	–					
8	Working status^b^	175	0.49	0.81	.27^c^	–				
9	Offline social support	175	3.78	0.60	.15^d^	.09	–			
10	Psychological help^b^	175	0.39	0.49	.13	.13	–.18^d^	–		
11	Depression	175	1.83	0.45	–.13	–.17^d^	–.36^c^	.24^c^	–	
12	Breast cancer-related concerns	175	2.64	0.55	–.09	–.19^d^	–.44^c^	.18^d^	.43^c^	–
13	Emotional well-being	175	3.53	0.77	.04	.20^c^	.32^c^	–.18^d^	–.56^c^	–.49^c^

^a^Standardized into Z scores.

^b^Coded 0=no, 1=yes.

^c^Correlations significant at the .01 level.

^d^Correlations significant at the .05 level.

### Depression

Regression results (Table 5) indicated a main effect of working status, psychological help, offline social support, cognitive avoidance, and a marginally significant effect of emotional expression on depression. No main effects of the intensity of support group participation and emotional processing were found. The higher that patients scored on cognitive avoidance, the more depressive feelings they reported. In contrast, emotional expression was negatively related to depression. Furthermore, an interaction effect of emotional expression and intensity of support group participation was found.

**Figure 1:**
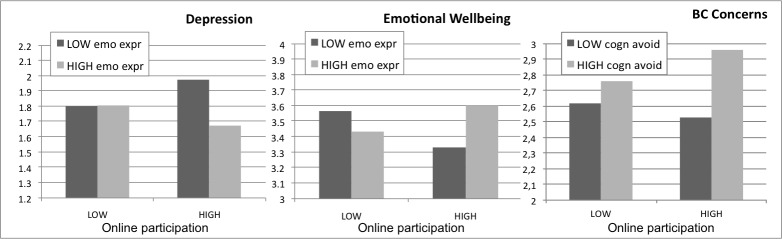
Interactions of the intensity of online support group participation and coping style on depression, emotional well-being, and breast cancer-related concerns (psychological well-being: Y-axis shows the absolute score; intensity of online participation and coping style: Low=1 standard deviation below the mean; High=1 standard deviation above the mean).

For patients expressing their emotions, intensity of online support group participation was associated with less depressive feelings. In contrast, for patients who scored low on emotional expression, online activity was associated with more depressive feelings (Figure 1). No other interaction effects on depression were observed.

**Table 5:** Hierarchical regression results for the effects of support group participation and coping style on depression, emotional well-being, and breast cancer–related concerns.

			Depression (n=162)					Emotional well-being (n=141)				Breast cancer concerns (n=162)				
Variable			b (SE)	ß		*P*		b (SE)	ß	*P*		b (SE)		ß		*P*
**Step 1: Covariates**																
	Working status^a^	–0.13 (0.06)		–.14		.047		0.20 (0.11)	.13		.072		–0.22 (0.07)	–.20		.003
	Psychological help^a^	0.14 (0.07)		.15		.039		–0.14 (.12)	–.88		.245		0.10 (0.08)	.09		.205
	Offline social support	–0.10 (0.03)		–.23		.002		0.11 (0.06)	.15		.045		–0.22 (0.04)	–.42		<.001
	Illness stage	–		–		–		–0.13 (0.06)	–.17		.023		–	–		–
	Age	–		–		–		–	–		–		–0.14 (0.04)	–.25		<.001
**Step 2: Main effects**																
	(A) Support group participation	0.01 (0.03)		.02			.748	–0.02 (0.06)	–.02		.778		0.03 (0.04)	.05		.459
	(B) Cognitive avoidance	0.13 (0.03)		.28			.000	–0.37 (0.06)	–.47		<.001		0.14 (0.04)	.27		<.001
	(C) Emotional processing	0.05 (0.04)		.11			.220	–0.07 (0.07)	–.09		.312		–0.07 (0.05)	–.12		.159
	(D) Emotional expression	–0.07 (0.04)		–.16			.067	0.04 (0.07)	.05		.603		0.05 (0.04)	.09		.290
**Step 3: Interaction effects**																
	A x B	–0.03 (0.03)		–.08			.273	–0.02 (0.05)	–.03		.709		0.07 (0.03)	.15		.027
	A x C	–0.01 (0.04)		–.02			.856	0.09 (0.07)	.11		.167		–0.03 (0.04)	–.05		.529
	A x D	–0.08 (0.04)		–.17			.030	0.10 (0.06)	.14		.089^b^		–0.02 (0.04)	–.03		.671
	*R* ^*2*^	.31						.39					.41			
	ANOVA	*F*_10,151_=6741					<.001	*F*_11,129_=7500			<.001		*F*_11,150_=9309			<.001
	Adjusted *R* ^*2*^	.26						.34					.36			
	Cohen’s *f* ^2^	0.45						0.64					0.69			

^a^Coded 0=no, 1=yes.

^b^This marginally significant effect may be due to a smaller sample (n=141) caused by incomplete scores regarding the covariate *illness stage*; the interaction is significant without the inclusion of *illness stage*, b(SE)=0.17(.07), *ß=.*17, **P*=.*02, n=162.

### Emotional Well-Being

Offline social support, illness stage, and cognitive avoidance showed a main effect on emotional well-being. No main effects of intensity of online support group participation and the other coping styles were found. Again, the more patients avoided thoughts, the worse their sense of well-being. Furthermore, a marginally significant interaction between emotional expression and support group participation was found (Table 4).

Results tentatively suggest that for patients low on emotional expression, being more active in an online support group was negatively associated with emotional well-being. For patients who expressed their emotions, the intensity of online support group participation was positively related to emotional well-being (Figure 1). No other interaction effects were observed on emotional well-being.

### Breast Cancer–Related Concerns

Results showed a main effect of working status, offline social support, age, and cognitive avoidance. No main effects of intensity of online participation or the other coping styles were found. Additionally, an interaction between cognitive avoidance and support group participation on breast cancer–related concerns was found (Table 4). Patients who were rather active online and tried to avoid thoughts about their illness had more breast cancer–related concerns than active patients who scored low on cognitive avoidance (Figure 1). No other interaction effects were found.

## Discussion

### Principal Findings

The present cross-sectional study tested the interaction of online support group participation and coping styles concerning illness-related thoughts and emotions on psychological well-being. The expected interaction was found on several occasions. Specifically, results suggest that patients coping with their illness by expressing their emotions may benefit more from online support group participation than patients who approach or acknowledge their emotions less. For patients who approached their emotions, active participation was positively related to emotional well-being and negatively related to depression. For patients who scored low on emotional expression, however, active participation was related to higher levels of depression and lower levels of emotional well-being. Finally, for patients who were more avoidant, the intensity of online support group participation was related to higher levels of breast cancer–related concerns, compared to patients who were less avoidant. These findings support the assumption that the relationship between online support group participation and well-being might be influenced by individual differences in coping styles. The current results suggest that active online support group participation may be more beneficial for individuals expressing their thoughts and emotions.

A potential explanation for the negative relation between the intensity of online participation and breast cancer-related concerns among patients with a more avoidant coping style is that these patients may be less able to cope with the negative content on online forums; they may be overwhelmed by the sad and frightening stories from patients in the same condition. In some online support group interview studies, patients mentioned having difficulties being confronted with negative sides of the disease [21,48], and some even withdrew to avoid painful details about cancer [49]. Previous research also showed that breast cancer patients use optimistic stories from peers as a source for inspiration [50]. Patients with an avoidant coping style may search for such positive stories but at the same time encounter negative stories they cannot cope with. Alternatively, patients with more concerns might be more active support seekers, but in turn also become more avoidant to be able to deal with their own extreme emotions and potentially distressing stories from online peers. Sometimes avoiding thoughts and emotions might be beneficial in order to prevent becoming overwhelmed with negative information from others. Since this study has a cross-sectional design, we cannot draw conclusions on the direction of the relationships we found. Therefore, there is a need for longitudinal studies on peer-led support groups, including patients’ coping styles. In addition, future research should focus on the content to reveal more insight into support group participation of patients with different coping styles, especially avoidant patients.

The present findings further previous research by showing that individual coping differences among online support seekers are likely to influence the relationship between online support group participation and psychological well-being. The current study might explain null findings in previous studies [11,12]. We found no direct relation between the intensity of participation and well-being, but we did find interactions of coping style and online participation on well-being. Support group participation may intensify certain positive processes that are already present in patients, such as the expression of emotions, but may also influence patients negatively when adaptive coping styles are less present. It is important to take these personal factors into account when we investigate the effectiveness of online support groups. Recently, studies have started looking into individual differences that may influence the effects of online support group participation. For example, a study showed different effects on emotional well-being depending on patients’ level of health self-efficacy [51].

Our findings also showed that other factors, such as illness stage and offline social support, were sometimes more related to psychological well-being than coping style or the interaction of support group participation and coping style. This underscores the importance of including “offline” factors that affect patients’ well-being when examining online support group effectiveness. Illness characteristics are often considered, but other factors outside the online support group that may influence well-being, such as support from friends and relatives or professional psychological help are often left out. Researchers should be careful not to exaggerate the effects of online peer support and distinguish effects directly caused by online participation, effects caused by other (offline) factors, and factors that could be strengthened by online support group participation (such as certain coping strategies). Future studies should also further examine the strength of these effects, as the effect sizes observed in the present research were rather modest.

### Limitations and Future Research

A limitation of this research is the cross-sectional design, which warrants caution in interpreting the direction of causality. A longitudinal study is needed to test causal relationships. For example, it is possible that not being able to express one’s emotions negatively affects well-being and that this decreased well-being, in turn, prompts patients to become more active online in order to find support. From a modeling or skills perspective [52], active participation in online support communities may help patients with more repressive coping styles to learn over time how to positively approach their illness and express their emotions. The latter effects are more likely to surface in longitudinal studies that track patients over an extended period of time. Future studies should therefore test the causal directions of the presently observed relationship and also examine whether patients becoming more active online can be beneficial for patients with repressive coping styles in the longer run.

Furthermore, no interactions of emotional processing and online participation on psychological well-being were found. This can be explained by the suggestion of Stanton and colleagues [30] that the effect of emotional processing depends on its contribution to emotional expression. When thoughts and feelings are not expressed, they may become ruminative, which may negatively affect well-being. Considering that the effectiveness of approaching emotions depended on the expression of emotions—and not on processing emotions—the scale measuring emotional expression may be a more important determinant of well-being. Future research should look further into the interrelations between different, yet related, coping styles.

Finally, knowledge in this field may be extended by studying differences between forum users and non-users. Questions of interest include which patients decide to participate in these online support groups and whether non-users should at any time be encouraged to participate.

### Conclusion

The current findings tentatively suggest that the effectiveness of online peer support is partly influenced by individual differences in coping style regarding thoughts and emotions. Patients who actively coped with their emotions and thoughts and participated actively within an online support community reported a better sense of psychological well-being than online active patients who coped less actively with their emotions. Although the current findings are cross-sectional, one plausible interpretation might be that patients who actively approach their emotions may benefit most from online support group participation, because online writing may reinforce the effectiveness of active coping styles. Since we found no direct relation between the intensity of online participation and psychological well-being, but several interactions between online activity and coping styles on well-being, patients’ initial coping abilities should be taken into account when examining the effectiveness of online peer support in future research.

## Abbreviations

CES-D: Center for Epidemiological Studies—Depression Scale
FACIT: Functional Assessment of Chronic Illness Therapy
SE: standard error
